# Lipin-1-derived diacylglycerol activates intracellular TRPC3 which is critical for inflammatory signaling

**DOI:** 10.1007/s00018-021-03999-0

**Published:** 2021-11-10

**Authors:** Javier Casas, Clara Meana, José Ramón López-López, Jesús Balsinde, María A. Balboa

**Affiliations:** 1 Instituto de Biología y Genética Molecular, Consejo Superior de Investigaciones Científicas (CSIC), Valladolid, Spain; 2 Departamento de Bioquímica y Biología Molecular y Fisiología, Facultad de Medicina, Universidad de Valladolid, 47003 Valladolid, Spain; 3 Centro de Investigación Biomédica en Red de Diabetes y Enfermedades Metabólicas Asociadas (CIBERDEM), 28029 Madrid, Spain

**Keywords:** TRPC3, Lipin-1, Macrophages, Inflammation, DAG, Ca^2+^ release

## Abstract

Exposure to Gram-negative bacterial LPS exacerbates host immune responses and may lead to sepsis, a life-threatening condition. Despite its high mortality and morbidity, no drugs specifically directed to treating sepsis are currently available. Using human cell genetic depletion, pharmacological inhibition, live-cell microscopy and organelle-targeted molecular sensors we present evidence that the channel TRPC3 is activated intracellularly during macrophage exposure to LPS and is essential for Ca^2+^ release from internal stores. In this manner, TRPC3 participates in cytosolic Ca^2+^ elevations, activation of the transcription factor NF-κB and cytokine upregulation. We also report that TRPC3 is activated by diacylglycerol generated by the phosphatidic acid phosphatase lipin-1. In accord with this, lipin-1-deficient cells exhibit reduced Ca^2+^ responses to LPS challenge. Finally, pharmacological inhibition of TRPC3 reduces systemic inflammation induced by LPS in mice. Collectively, our study unveils a central component of LPS-triggered Ca^2+^ signaling that involves intracellular sensing of lipin-1-derived DAG by TRPC3, and opens new opportunities for the development of strategies to treat LPS-driven inflammation.

**Supplementary Information:**

The online version contains supplementary material available at 10.1007/s00018-021-03999-0.

## Introduction

Receptors on the surface of innate immune cells recognize multiple molecules derived from pathogens. These receptors initiate an inflammatory reaction that helps to eliminate the pathogen and repair the damaged area. TLR4, a prominent member of the Toll-like receptor family, is the canonical receptor for lipopolysaccharide (LPS) from Gram-negative bacteria [7]. LPS recognition by TLR4 initiates a cascade of events that culminates in the activation of the transcription factors AP-1, NF-κB, or IRF-3 through different signaling branches [7]. The final outcome is the transcriptional upregulation of cytokines, enzymes and proteins that help inactivate the invading microorganism. However, excessive TLR4 activation orchestrated by elevated concentrations of LPS could be detrimental, leading to sepsis, a life-threatening organ dysfunction caused by the dysregulated host response to infection. Hence, a finer knowledge of TLR4-mediated signaling is key to find helpful targets for the management of infections and inflammatory diseases.

It is now clearly established that complete TLR4 responses require cellular Ca^2+^ fluxes and [Ca^2+^]i elevations [10, 26, 40, 43, 49]. Ca^2+^ signaling in immune cells has traditionally been considered to be due to the release of Ca^2+^ from internal stores, such as the endoplasmic reticulum (ER), followed by Ca^2+^ influx through plasma membrane channels to restore homeostasis [12]. This mechanism is known as store-operated Ca^2+^ entry (SOCE). Regarding LPS-induced Ca^2+^ fluxes, it has been proposed that they are initiated by the activation of phospholipase Cγ2 (PLCγ2) at the plasma membrane, mediated by the TLR4 co-receptor CD14 [10, 49]. This model assumes that PLCγ2–derived inositol 1,4,5-trisphosphate (InsP_3_) interacts with receptors at the ER which mediate Ca^2+^ release. As a consequence, SOCE mechanisms are activated and participate in the [Ca^2+^]_i_ rise. The discovery that elimination of the proteins that regulate SOCE through interaction with ORAI channels at the plasma membrane, i.e. STIM1 and STIM2, has no discernible effect on LPS signaling in macrophages [45], has encouraged research programs to identify alternative players in Ca^2+^ dynamics.

Some members of the transient receptor potential canonical (TRPC) channel superfamily can be activated by second messengers of lipid nature. TRPC3, together with TRPC6 and TRPC7, are calcium-permeable nonselective cation channels that share the special attribute of being activated by diacylglycerol (DAG) [17, 19]. Of these, TRPC3 is the best characterized in innate immune cells [23]. While initially described to be involved in SOCE, TRPC3 possesses the capacity to directly bind DAG thanks to a lateral fenestration in its pore domain which is critical for channel gating [15, 27]. It is thought that DAG binding to TRPC3 constitutes the key event that drives TRPC3 activation and channel-mediated Ca^2+^ signaling. DAG may be transiently generated during signaling through the action of PLCs on membrane phosphoinositides, but it may also be produced in a long lasting manner by other means, involving the concerted action of several enzymes and pathways [2, 5, 18]. We have recently described the generation of DAG by the phosphatidic acid phosphatase, lipin-1, during macrophage activation by LPS [33]. Lipin-1 is a prominent member of a family of phosphatidic acid (PA) phosphatase enzymes with key roles in metabolism and signaling [5, 30, 50]. In the absence of lipin-1 many TLR4-mediated responses are altered in macrophages, including cytokine production, ultimately affecting inflammation in animal models of disease [33, 34]. Thus, lipin-1-derived DAG is of great importance during LPS challenge, albeit its downstream direct targets are poorly defined.

In this work, we have identified a novel signaling role for TRPC3 during LPS activation of primary human macrophages and related cell lines. We demonstrate that TRPC3 is activated by lipin-1-derived DAG during LPS activation, and that blockade of TRPC3 activity ameliorates LPS-driven inflammation and sepsis development in animal models.

## Results

### Involvement of TRPC3 channels in LPS-induced cytokine production

**Fig. 1:**
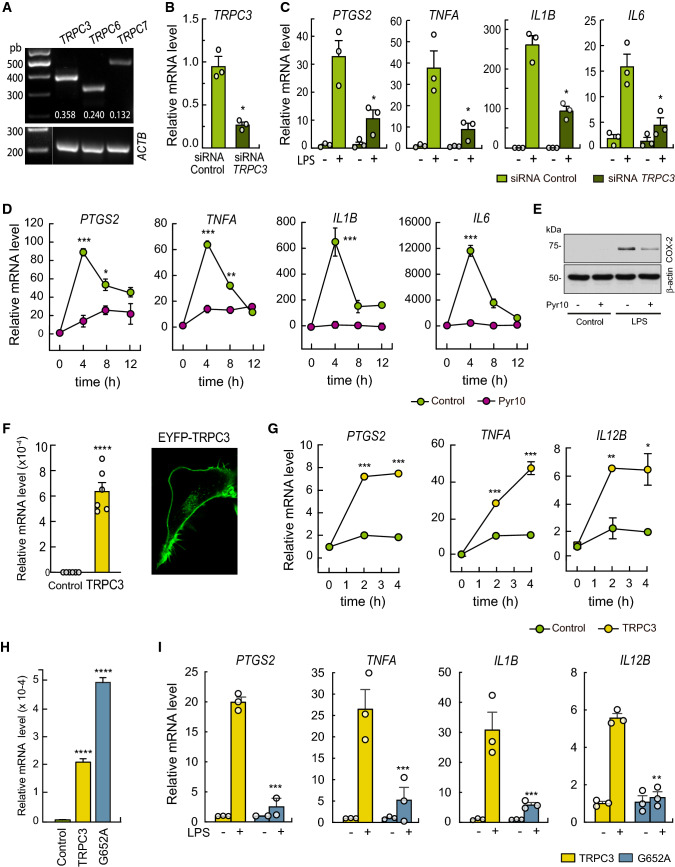
TRPC3 is required for LPS-mediated upregulation of proinflammatory genes. **A** Semi-quantitative PCR analysis of the expression of DAG-sensitive TRPC channels in THP-1 macrophages. Data quantification using *ACTB* for normalization is shown in white. **B** THP-1 macrophages were silenced with control siRNAs or specific siRNAs for *TRPC3*. *TRPC3* mRNA levels were quantified by qPCR using *ACTB* as the reference gene. Error bars represent SEM (*n* = 3). **C** Silenced THP-1 macrophages were treated with 100 ng/ml LPS for 5 h and mRNA levels for the indicated genes were quantified by qPCR. Changes in mRNA levels relative to untreated cells are represented. Error bars represent SEM (*n* = 3). **D** THP-1 macrophages were pretreated with 10 μM Pyr10 for 30 min and then treated with 100 ng/ml LPS for the indicated times. mRNA levels for the indicated genes were analyzed by qPCR. Error bars represent SEM (*n* = 3). *, *p* < 0.05; **, *p* < 0.01, ***, *p* < 0.001; Student’s *t* test. **E** THP-1 macrophages were pretreated with 10 μM Pyr10 for 30 min and then treated with 100 ng/ml LPS for 4 h. COX-2 levels were evaluated by immunoblot using specific antibodies. β-actin was used as the loading control. **F**–**I**) HEK-TLR4 cells stably overexpressing TRPC3, the mutant G652A-TRPC3, or an empty plasmid (control cells) were stimulated with 1 μg/ml LPS for the indicated periods of time (**G**), or 3 h (**I**) and mRNA levels of the indicated genes analyzed by qPCR (**G**, **I**). Changes in *TRPC3* mRNA levels were quantified by qPCR (left panel) and fluorescence from eYFP-TRPC3 observed by confocal microscopy (right panel). Scale bar represents 10 μm (**F**). Changes in *G652*-*TRPC3* mRNA levels were quantified by qPCR (**H**). Error bars represent SEM (*n* = 3). *, *p* < 0.05; **, *p* < 0.01, ***, *p* < 0.001; ****, *p* < 0.0001, by Student’s *t* test

We began this study by analyzing the expression of DAG-regulated TRPC channels in human THP-1 macrophages using conventional semi-quantitative PCR. As shown in Fig. 1, TRPC3 is expressed in these cells, as well as TRPC6 and 7 (Fig. 1A). Interestingly, reduced expression levels of TRCP3 by siRNA or use of the TRPC3 cell-permeable selective blocker Pyr10 [41] strongly blunted the LPS-induced upregulation of a number of proinflammatory genes, including *PTGS2*, *TNFA*, *IL1B* and *IL6* (Fig. 1B–D). The increased expression of COX-2 protein (*PTGS2*) induced by LPS was also inhibited by Pyr10 (Fig. 1E). Consistent with these data, overexpression of the TRPC3 channel in HEK293 cells expressing the human sequences for TLR4/MD2/CD14 (HEK-TLR4 cells), markedly increased the expression levels of *PTGS2*, *TNFA*, and *IL12B* after treatment with LPS, as compared to control cells (Fig. 1F, G).

We investigated next whether DAG was required for TRPC3 to regulate the upregulation of inflammatory genes mediated by LPS. To this end, we took advantage of the recent finding that a single mutation in TRPC3, G652A, blunts its capacity to recognize DAG [27]. We transfected the HEK-TLR4 cells with the G652A-TRPC3 mutant and studied the capacity of the cells to produce inflammatory effectors. The results indicated a reduced capacity of G652A-TRPC3-transfected cells to upregulate *PTGS2, TNFA, IL1B,* and *IL12B* compared with cells transfected with the wild-type TRPC3 construct after LPS treatment (Fig. 1H, I). Collectively, these results suggest that DAG recognition by TRPC3 is key for LPS-activated inflammatory responses.

### LPS-induced calcium signaling is mediated by TRPC3

**Fig. 2:**
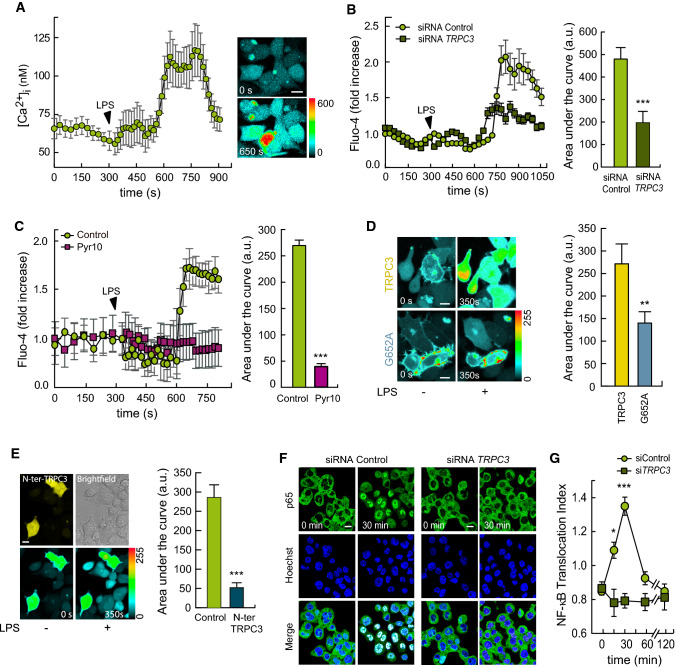
LPS-induced Ca^2+^ fluxes and signaling depend on TRPC3. **A** THP-1 macrophages were labeled with Fluo-4, and fluorescence was recorded before and after treating the cells with 100 ng/ml LPS, as indicated. Absolute changes in [Ca^2+^]i are shown (*n* = 25 cells) (left panel). Representative pictures showing fluorescence intensities at 0 s and 350 s after LPS treatment are shown (right panel). Scale bar represents 10 μm. **B** THP-1 macrophages silenced with siRNA control or against *TRPC3* were treated as in **A** Relative changes in [Ca^2+^]i are shown (left panel). Quantification of the area under the curve is shown in arbitrary units (a.u., right panel). Error bars represent SEM (siRNA Control, *n* = 28; siRNA *TRPC3*, *n* = 23). ***, *p* < 0.001, Student’s *t* test. **C** Macrophages were treated as in **A** and changes in [Ca^2+^]i were analyzed in the absence or presence of 10 μM Pyr10 (left panel). Quantification of the area under the curve is shown in arbitrary units (right panel). Error bars represent SEM (*n* = 25) ***, *p* < 0.001, Student’s *t* test. **D** HEK-TLR4 cells were transfected with TRPC3 or the G652A-TRPC3 mutant, labeled with Fluo-4, and fluorescence was recorded before and after treatment with 1 μg/ml LPS. Images of [Ca^2+^]i peak time changes are shown (left panels). Quantification of the area under the curve is shown in arbitrary units (right panel). Error bars represent SEM (*n* = 20). **, *p* < 0.01, by Student’s *t* test. **E** HEK-TLR4 cells transiently transfected with a dominant negative N-terminal fragment of TRPC3 (eYFP-N-ter-TRPC3, yellow cells, left panel, upper pictures) were loaded with Fluo-4 and fluorescence was recorded before and after treatment with 1 μg/ml LPS. Representative pictures showing Fluo-4 fluorescence intensities at 0 s and 350 s after LPS treatment are shown (left panel, bottom pictures). Analysis of relative changes in [Ca^2+^]i were performed and quantification of the areas under the curve (a.u., right panel) from negative (control) and positive eYFP-N-ter-TRPC3 (N-ter TRPC3) cells are shown. Scale bar represents 10 μm. Error bars represent SEM (*n* = 22). ***, *p* < 0.001, by Student’s *t* test. **F**, **G**) THP-1 macrophages were silenced with control siRNAs or specific siRNAs for *TRPC3*, treated with 100 ng/ml LPS for the indicated periods of time and immunostained using antibodies against p65 NF-κB. Nuclei were counterstained with Hoechst 33342. Confocal microscopy pictures (**F**) and NF-κB nuclear translocation indexes (**G**) are shown. Error bars represent SEM (*n* = 3 images with 75 and 100 cells/image). Scale bar represents 10 μm. *, *p* < 0.05; ***, *p* < 0.001, by Student’s *t* test

Elevations in intracellular calcium ([Ca^2+^]i) during activation by LPS constitute an obligate signaling step [10, 26, 40, 43, 49]. In the next series of experiments, we used live-cell imaging to explore the involvement of TRPC3 in the regulation of Ca^2+^ fluxes during LPS activation. Using the dynamic single-wavelength fluorescent Ca^2+^ indicator Fluo-4, whose increase in fluorescence intensity reflects a rise in the cytoplasmic Ca^2+^ level, we detected significant [Ca^2+^]i increases after treating the human THP-1 macrophages with LPS (Fig. 2A, B), in agreement with previous reports [26, 40, 49]. Importantly, macrophages deficient in TRPC3 exhibited smaller [Ca^2+^]i rises than control cells (Fig. 2B). Quantification of the areas under the curves demonstrated that, in the absence of TRPC3, cells experienced a reduction in [Ca^2+^]i of ~60% during activation. To further assess the role of TRPC3 in intracellular Ca^2+^ raises, we used several strategies, namely treating the macrophages with Pyr10, and overexpressing the G652A-TRPC3 mutant or an N-terminal dominant negative fragment of TRPC3 (N-ter-TRPC3) in HEK-TLR4 cells [38]. All these strategies were found to strongly reduce the LPS-induced [Ca^2+^]i increases respect to control cells (Fig. 2C-E).

NF-κB is a transcription factor implicated in the upregulation of many of the genes described above, and its full activation depends on Ca^2+^ fluxes [40, 43]. Thus, we studied next the activation of this transcription factor by analyzing the translocation to the nucleus of one of its classical subunits, p65. LPS treatment of THP-1 macrophages induced a time-dependent translocation of p65 to the nuclear compartment. Such translocation reached a maximum at 30 min after LPS exposure, and was markedly reduced in TRPC3-deficient macrophages (Fig. 2F, G**)**. Collectively, these data indicate that TRPC3 is involved in Ca^2+^ fluxes and in the activation of transcription factors that participate in the upregulation of inflammatory cytokines by LPS.

### Plasma membrane TRPC3 currents are not affected by LPS

**Fig. 3:**
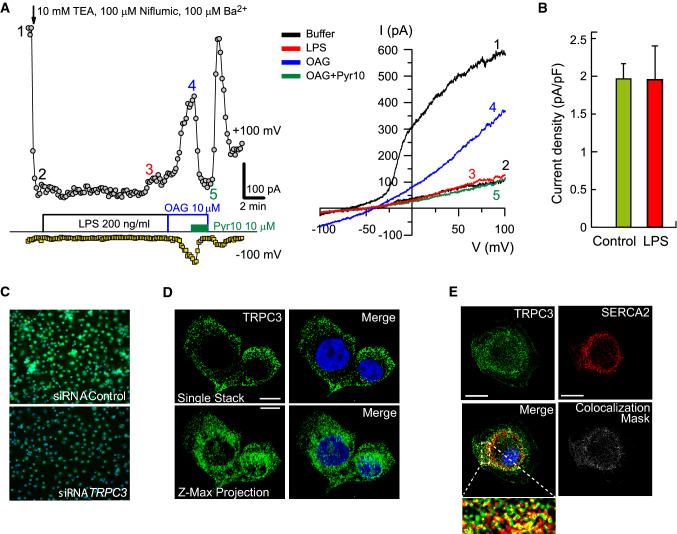
DAG drives TRPC3-dependent inflammatory activation not at the plasma membrane. **A** Typical recordings of whole-cell currents obtained at − 100 and + 100 mV in peritoneal macrophages. Left panel shows time courses of current densities recorded in every ramp at + 100 mV (open circles) and −100 mV (open squares). After blocking unwanted currents with 10 mM TEACl 100 μM niflumic acid, and 100 μM BaCl_2_, the cell was stimulated with 200 ng/ml LPS, 10 μM OAG or OAG plus 10 μM Pyr10 as indicated. Whole I/V curves recorded at times labeled from 1 to 5 are depicted in the right graph. **B** Average Pyr10-sensitive current densities obtained at − 100 mV after 10 min of stimulation with 200 ng/ml LPS (*n* = 5 cells) or after 10 min of recording in control solution (*n* = 5 cells). **C** HMDM were treated with control siRNAs or specific siRNAs for *TRPC3*, fixed and stained with specific antibodies against TRPC3 (green). Nuclei were counterstained with Hoechst 333342 (blue). **D** THP-1 macrophages were immunostained with antibodies against TRPC3 (green) and counterstained with Hoechst 333342 (nuclei, blue). Images show analyses of fluorescence by confocal microscopy (top). Images of maximum z-projection analyses of 22 stacks are also shown (bottom). Scale bar represents 10 μm. **E** HMDM were fixed and stained with antibodies against TRPC3 (green), or SERCA2 (red) and counterstained with Hoechst 333342 (nuclei, blue). Merge fluorescence and colocalization mask is also shown. The bottom image is a detailed amplification of the framed zone from the merge image. Scale bar represents 10 μm

To investigate further the involvement of TRPC3 in signaling events during cellular treatment with LPS, we carried out whole-cell patch-clamp recordings. The advantage of this technology is that it can unequivocally measure the activation of channels located in the plasma membrane. Based on the studies measuring [Ca^2+^]i (Fig. 2) and in the biology of the TRPC3 channel [44], we anticipated that a significant inward current during treatment of the cells with LPS would be detected, reflecting the activation of ion currents through the channel. However, we failed to detect such activation (Fig. 3A, B). As a positive control of the experiment, we treated the macrophages with the synthetic DAG 1‐oleyl‐2-acetyl‐*sn*‐glycerol (OAG) to activate the TRPCs that could be present at the plasma membrane [19]. Upon addition of OAG, we did detect the activation of a Pyr10-sensitive current (Fig. 3A). These results show that macrophages express channels whose currents can be activated by DAG (TRPCs), and inhibited by Pyr10 (TRPC3) but, interestingly, these channels are not sensitive to LPS, at least at the plasma membrane.

Although TRPC3 channels have been localized to the plasma membrane, they may also be present in intracellular membranes, particularly those of the ER and mitochondria [16, 28]. Therefore, because of the results obtained in the patch-clamp experiments, we performed immunofluorescence measurements in human macrophages to clearly establish the localization of TRPC3 in these cells. We used validated commercially available TRPC3 antibodies that produced only marginal fluorescence in TRPC3-silenced macrophages (Fig. 3C). TRPC3 was mostly found in cytoplasmic membranes, being especially concentrated in membranes close to the nucleus. This was particularly evident when all the images obtained from single stacks were subjected to z-maximum projection (Fig. 3D). Colocalization experiments using antibodies against the ER membrane residing protein SERCA2 confirmed that most of the membranes where TRPC3 is located are part of the ER (Fig. 3E). In TRPC3-overexpressing HEK-TLR4 cells, the channel was also confirmed to be present in intracellular membranes resembling those of the ER (Fig. 1F).

### LPS increases DAG levels in the ER

**Fig. 4:**
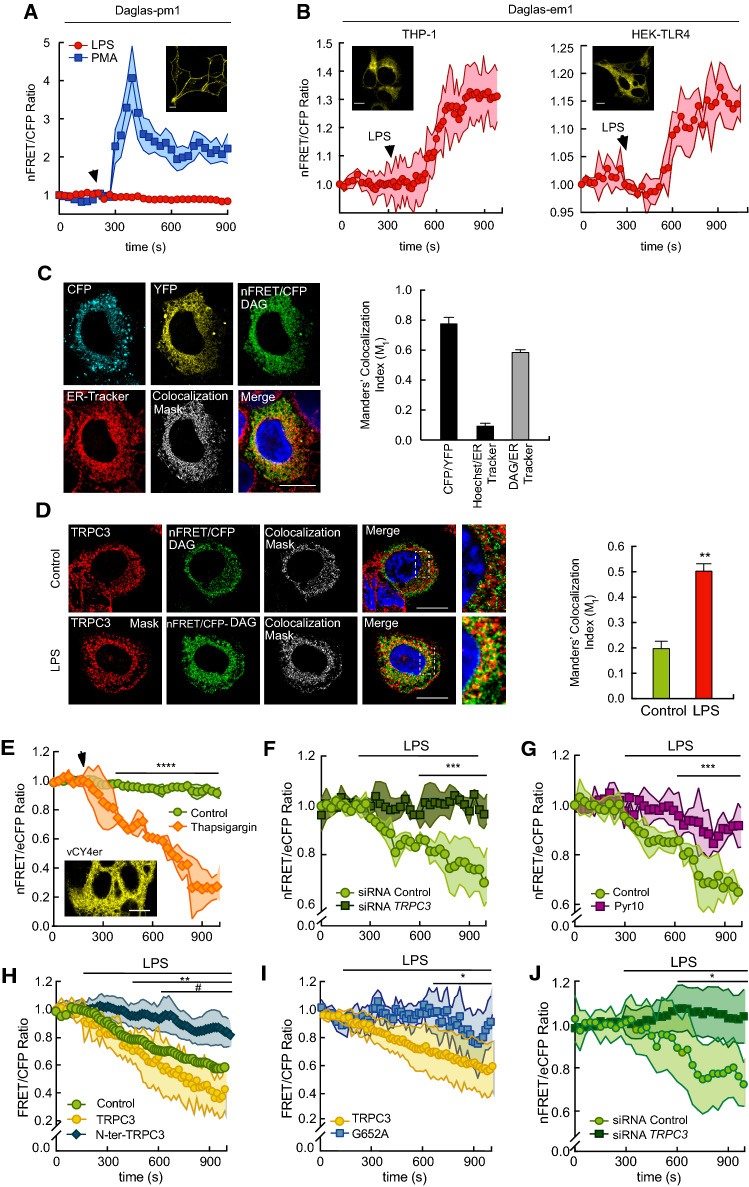
TRPC3 colocalizes with DAG at the ER and participates in Ca^2+^ release from the ER during LPS activation. **A** HEK-TLR4 cells transfected with the sensor Daglas-pm1 were stimulated with 1 μg/ml LPS (red circles) or 1 μM PMA (blue squares), as indicated. Time course changes in nFRET/CFP ratio are shown. Insert shows representative images of sensor fluorescence. Scale bar represents 10 μm. **B** THP-1 macrophages (left panel) and HEK-TLR4 cells (right panel), transfected with the sensor Daglas-em1, were stimulated with 100 ng/ml and 1 μg/ml LPS, respectively. Time courses of the changes in nFRET/CFP ratio are shown. Shadows in pink represent SEM (*n* = 27). Inserts show representative images of sensor fluorescence. Scale bar represents 10 μm. **C** THP-1 macrophages transfected with Daglas-em1 sensor were stained with 200 nM ER-Tracker and Hoechst 33342, and stimulated with 100 ng/ml LPS for 10 min. Fluorescence from CFP (cyan), YFP (yellow), nFRET/CFP (DAG, green), Hoechst 33342 (nuclei, blue), ER-Tracker (ER, red), was analyzed by confocal microscopy and images, including merge and the colocalization mask between DAG and ER-Tracker fluorescences (white), are shown (left panel). Scale bar represents 10 μm. Manders’ colocalization indexes (M_1_) are shown (right panel). Error bars represent SEM (*n* = 28). **D** THP-1 macrophages, transfected with the Daglas-em1 sensor, were treated or not with 100 ng/ml LPS for 5 min, fixed and stained with antibodies against TRPC3 (red) and Hoechst 33342 (blue). Fluorescence images analyzed by confocal microscopy are shown. Images on the right are a detailed amplification of the framed zones from the merge images (left panel). Scale bar represents 10 μm. Manders’ colocalization indexes (M_1_) between DAG (nFRET/CFP, green) and TRPC3 (red) in cells stimulated or not with LPS are shown (right panel). Error bars represent SEM (*n* = 22 cells). **, *p* < 0.01; Student’s *t* test. **E**–**I**) HEK-TLR4 cells were transfected with the vCY4er calcium sensor, stimulated with 1 μg/ml LPS as indicated and fluorescence was analyzed by confocal microscopy. Mean nFRET/eCFP ratio ± SEM (shadow) is represented. (**E**) Cells were treated or not with thapsigargin (1 µM) (*n* = 40 cells). The inset shows representative vYC4er fluorescence images. Scale bar represents 10 μm. (**F**) Cells were treated with siRNA control or against *TRPC3* (*n* = 38 cells). (**G**) Cells were stimulated in the absence or presence of 10 μM Pyr10 (*n* = 42 cells). (**H**) Cells were transfected with TRPC3, the dominant negative N-ter-TRPC3 or an empty plasmid (Control) (*n* = 26 cells). **I** Cells were transfected with TRPC3 or the G652A-TRPC3 mutant (*n* = 32 cells). **J** HMDM were treated with siRNA control or against *TRPC3* and stimulated with 200 ng/ml LPS as indicated (*n* = 27 cells). Multiple t test was applied in **E**–**J**. *, *p* < 0.05; **, *p* < 0.01; ***, *p* < 0.001, ****, *p* < 0.0001. Panel **H**: *, N-ter-TRPC3 vs Control cells; #, N-ter-TRPC3 vs. TRPC3 transfected cells

To assess whether TRPC3 could be activated by DAG in endomembranes, we analyzed physiological DAG dynamics. We used genetically encoded FRET-based DAG biosensors that can be targeted specifically to different biological membranes, namely Daglas-pm1 and Daglas-em1. Daglas-pm1 is directed to the plasma membrane through an 11-aminoacid sequence of N-Ras that allows its prenylation [39]. Daglas-em1, directed to ER membranes, is obtained by mutating the C181 of N-Ras to Ser [39]. Transfected biosensors localized as expected in both THP-1 macrophages and HEK-TLR4 cells (Fig. 4A, B). HEK-TLR4 cells expressing Daglas-pm1 immediately showed a substantial increase in the intramolecular FRET/CFP ratio when stimulated with the DAG analog phorbol 12-myristate 13-acetate (PMA) (Fig. 4A). However, when the cells were stimulated with LPS, no substantial effect on the fluorescence emission ratio was detected, suggesting that this particular sensor is not detecting changes in DAG levels at the plasma membrane under these conditions. In contrast, cells expressing the ER-directed Daglas-em1 sensor showed a significant increase in FRET/CFP ratio after LPS stimulation in both THP-1 macrophages and HEK-TLR4 cells (Fig. 4B).

To precisely identify the endomembranes where the relevant DAG pool accumulates during LPS activation, THP-1 macrophages were transfected with the Daglas-em1 biosensor, stimulated with LPS, and subsequently labeled with the ER-specific marker ER-Tracker-TR (Fig. 4C). Colocalization between DAG and ER was studied using the Manders’ colocalization index M _1_ [31]. The colocalization index between FRET/CFP (representing DAG content) and ER-Tracker-TR (representing ER) was 0.58 ± 0.02. Given that the colocalization index between CFP and YFP—which were engineered into the same molecule—was 0.78 ± 0.04, these data suggest that a substantial amount of the DAG generated during macrophage stimulation with LPS accumulates in the ER.

### TRPC3 colocalizes with DAG at the ER and regulates [Ca^2+^]er during TLR4 activation

We investigated next whether the DAG generated in endomembranes upon LPS treatment localized within the same intracellular structures as TRPC3. Macrophages expressing the sensor Daglas-em1 were stimulated with LPS, immunostained with antibodies against TRPC3, and the colocalization between DAG and TRPC3 was analyzed. A marked increase in the colocalization index in stimulated cells compared with control cells in areas adjacent to the nucleus, where the ER is located, was appreciated (Fig. 4D). These results suggest that a significant amount of TRPC3 is found at the same structures where DAG accumulates, i.e. ER membranes, during LPS treatment.

Based on the findings that: (i) ion currents through TRPC3 are not activated at the plasma membrane by LPS, (ii) the channel mediates Ca^2+^ fluxes during activation, and (iii) the channel is present mainly in intracellular membranes in macrophages, we reasoned that TRPC3 could be involved in regulating the movement of Ca^2+^ from intracellular stores. To investigate this possibility, we took advantage of a FRET-based calcium cameleon indicator engineered to reside in the lumen of the ER (vYC4er) [35]. This indicator detects changes in [Ca^2+^]er during cellular stimulation [35]. HEK-TLR4 cells transfected with this indicator showed a reticular pattern of fluorescence characteristic of ER localization, and underwent a decrease in FRET/eCFP ratio when Ca^2+^ decreased in the organelle due to thapsigargin treatment (Fig. 4E). LPS treatment also reduced the FRET/eCFP ratio of the cameleon indicator (Fig. 4F-J). Interestingly, the effect was almost abolished when TRPC3-deficient cells were used (Fig. 4F, J) in the presence of Pyr10 (Fig. 4G), and also when the N-ter-TRPC3 dominant negative construct was overexpressed (Fig. 4H). Importantly, we corroborated these results in primary human monocyte-derived macrophages deficient in TRPC3 (Fig. 4J). These fluorescence changes reflect a reduction in [Ca^2+^]er during stimulation, and indicate that the TRPC3 channel plays a key role in the process. We also observed an enhanced reduction of the FRET/eCFP ratio when TRPC3 was overexpressed in LPS-treated cells, compared with control cells, but not when the DAG-binding mutant G652A was used (Fig. 4H, I). These results corroborate the positive effect of the channel in Ca^2+^ mobilization from the ER and the importance of DAG recognition in this effect. Taken together, these results support the view that, in macrophages, LPS treatment rises DAG levels in the ER, increases DAG/TRPC3 colocalization, and promotes Ca^2+^ release from the organelle in a TRPC3 activity-dependent manner.

### ER enrichment with DAG during LPS activation depends on Lipin-1

**Fig. 5:**
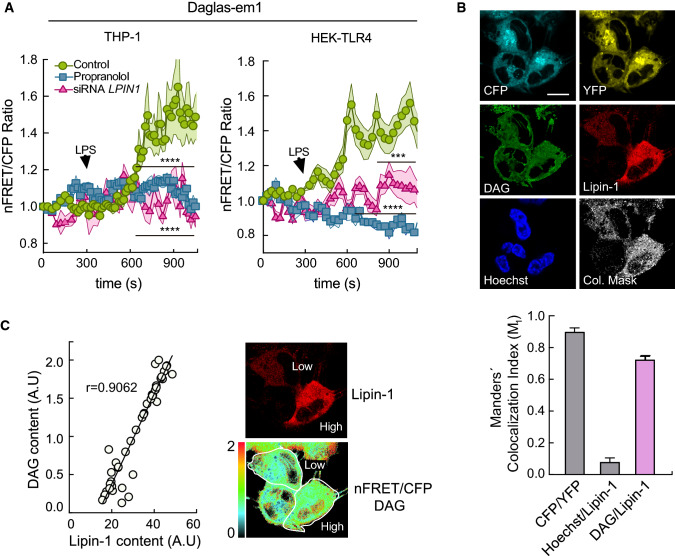
Lipin-1 mediates DAG increases in the ER during LPS activation. **A** THP- 1 macrophages (left panel) and HEK-TLR4 cells (right panel) were transfected with the Daglas-em1 sensor. Fluorescence was analyzed by confocal microscopy before and after treating the cells with LPS (100 ng/ml and 1 μg/ml LPS, respectively), in cells treated or not with 200 μM propranolol, or silenced for *LPIN1*, as indicated. Changes in nFRET/CFP ratio are shown. Multiple t test was applied. ***, *p* < 0.001, ****, *p* < 0.0001. **B** THP-1 macrophages transfected with the Daglas-em1 sensor and lipin-1- mCherry were stimulated with 100 ng/ml LPS for 10 min and counterstained with Hoechst 33342. Fluorescence from CFP (cyan), YPF (yellow), nFRET/CFP (DAG, green), lipin-1-mCherry (red) Hoechst 33342 (nuclei, blue), was analyzed by confocal microscopy and images, including the colocalization mask between DAG and lipin-1- mCherry fluorescences (white), are shown. Scale bar represents 10 μm. Manders’ colocalization indexes (M_1_) were calculated and are shown (lower panel). Error bars represent SEM (*n* = 22). **C** Cells expressing lipin-1-mCherry were individually analyzed for DAG content (nFRET/CFP fluorescence) and lipin-1-mCherry content (red fluorescence) and plotted against each other to obtain a regression index (*r* = 0.9062, *n* = 45 cells, left panel). Representative fluorescence images are shown in the right panel

Subsequent work was aimed to elucidate the molecular mechanisms by which DAG is generated in endomembranes during LPS stimulation. We have recently demonstrated that a member of the lipin family, called lipin-1, acts as a modulator of LPS-triggered signaling cascades [29, 33, 46]. Hence, we considered that lipin-1 could constitute a reasonable candidate to regulate DAG levels in the ER under those circumstances. As a first approach, we used propranolol, a well-established inhibitor of the enzymatic activity of lipins ([3, 4, 32] and estimated DAG production in the ER using the Daglas-em1 biosensor (Fig. 5A). The LPS-stimulated increase in the FRET/CFP ratio was strongly inhibited by propranolol, both in THP-1 macrophages and HEK-TLR4 cells. Moreover, use of lipin-1-deficient cells by siRNA silencing [33] demonstrated no significant increases in ER DAG levels during the LPS challenge (Fig. 5A**)**. In addition, high colocalization between the DAG present in the ER and lipin-1 was noted, as judged by the high M_1_ index found between the FRET/CFP ratio from the Daglas-em1 biosensor, and the fluorescence from the chimera lipin-1-mCherry (M_1_= 0.72 ± 0.02) (Fig. 5B). A very high positive correlation (*r*=0.9062) was also found between the levels of DAG in the ER (FRET/CFP ratio) and lipin-1-mCherry expression levels in transfected macrophages (Fig. 5c). These results demonstrate that lipin-1 and DAG are in close proximity in the ER—as it would be expected from an enzyme and its product—and that LPS-stimulated DAG production in the ER depends on lipin-1 expression.

### Lipin-1 participates in Ca^2+^ Fluxes and TRPC3-dependent cytokine production during LPS stimulation

**Fig. 6:**
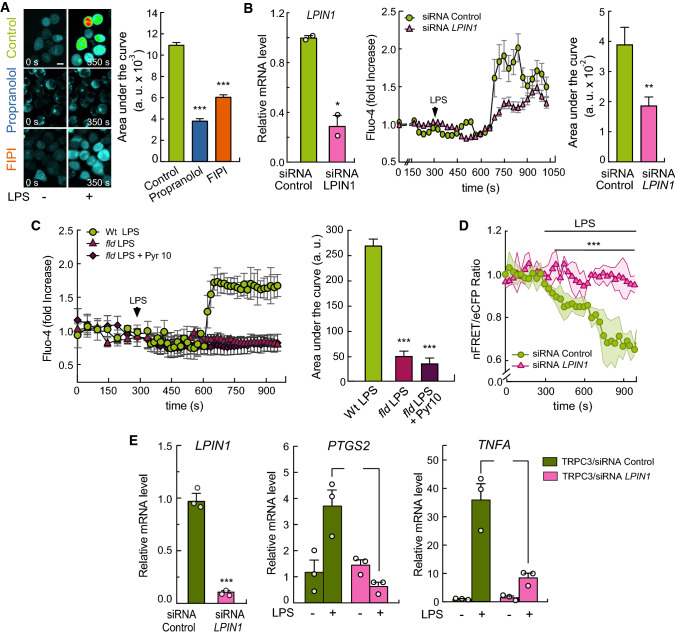
Lipin-1 participates in Ca^2+^ fluxes during LPS stimulation. **A** THP-1 macrophages were labeled with Fluo-4, and fluorescence was recorded before and after 100 ng/ml LPS treatment, in the absence or presence of 200 μM propranolol or 1 μM FIPI, as indicated. Representative fluorescence images (left panel) and quantification of the areas under the curves (arbitrary units, a.u.; right panel) are shown. Error bars represent SEM (*n* = 25). ***, *p* < 0.001, by Student’s *t* test. **B** THP-1 macrophages treated with siRNA control or against *LPIN1* were labeled with Fluo-4, and fluorescence was recorded before and after treating the cells with 100 ng/ml LPS, as indicated (middle panel). Quantification of the area under the curve is shown in arbitrary units (a.u., right panel). Error bars represent SEM (*n* = 25). mRNA levels for *LPIN1* analyzed by qPCR are also shown (left panel). Error bars represent SEM (*n* = 3). **, *p* < 0.01, Student’s *t* test. **C** Peritoneal macrophages from wt or lipin-1-deficient animals (*fld*) were used for [Ca^2+^]_i_ quantification as in B. Error bars represent SEM (*n* = 650). ***, *p* < 0.001, Student’s *t* test. **D** HEK-TLR4 cells were silenced with siRNA control, or against *LPIN1*, transfected with the vYC4er sensor, and changes in [Ca^2+^]_er_ were analyzed before and after treating the cells with 1 μg/ml LPS, as indicated. Mean nFRET/eCFP ratio ± SEM is represented (*n* = 40 cells). Multiple t test was applied. ***, *p* < 0.001. **E** HEK-TLR4 cells were transfected with TRPC3, silenced with siRNA control or against *LPIN1* and stimulated with 1 μg/ml LPS for 3 h. mRNA levels for the indicated genes were analyzed by qPCR. mRNA levels for *LPIN1* analyzed by qPCR is also shown (left panel). Error bars represent SEM (*n* = 3). **, *p* < 0.01; ***, *p* < 0.001, by Student’s *t* test

If, as the previous data suggested, lipin-1 is the enzyme that provides DAG for TRPC3 activation, inhibition or absence of the enzyme should affect the LPS-associated Ca^2+^ fluxes described above. We analyzed [Ca^2+^]i in macrophages treated with propranolol and found that the inhibitor prevented [Ca^2+^]i increases during cellular LPS treatment (Fig. 6A). The same effect was also observed in human macrophages silenced for *LPIN1* (Fig. 6B), and in murine macrophages with a spontaneous mutation in the *Lpin1* gene (*fld* animals), which abrogates lipin-1 expression [50] (Fig. 6C). Ca^2+^ responses of *fld* macrophages to LPS were not altered by Pyr10 treatment (Fig. 6C), supporting the idea that lipin-1 is upstream of TRPC3 activation in this setting.

[Ca^2+^]er was studied next using the calcium cameleon indicator vYC4er. The reduction in [Ca^2+^]er experienced by the cells during LPS treatment was significantly prevented in *LPIN1*-silenced cells (Fig. 6D). Further evidence for the implication of lipin-1 in TRPC3 activation was obtained when we assessed the upregulation of inflammatory genes in HEK-TLR4 cells that overexpressed TRPC3 and exhibited reduced levels of lipin-1 by gene silencing. The absence of lipin-1 clearly prevented TRPC3 from being involved in LPS-induced upregulation of *PTGS2* and *TNFA* (Fig. 6E).

The substrate of lipins, PA, can be produced in activated cells by the direct action of phospholipase D on membrane glycerophospholipids [2, 6]. We treated macrophages with the specific phospholipase D inhibitor FIPI  [42], and found that it affected the rise in [Ca^2+^]i during LPS treatment (Fig. 6A), consistent with a scenario whereby phospholipase D provides phosphatidic acid for DAG production and TRPC3 activation. Collectively, these results highlight a key role for lipin-1 in regulating Ca^2+^ mobilization from the ER and TRPC3-mediated inflammation during LPS activation.

### TRPC3 inhibition ameliorates systemic inflammation in vivo

**Fig. 7:**
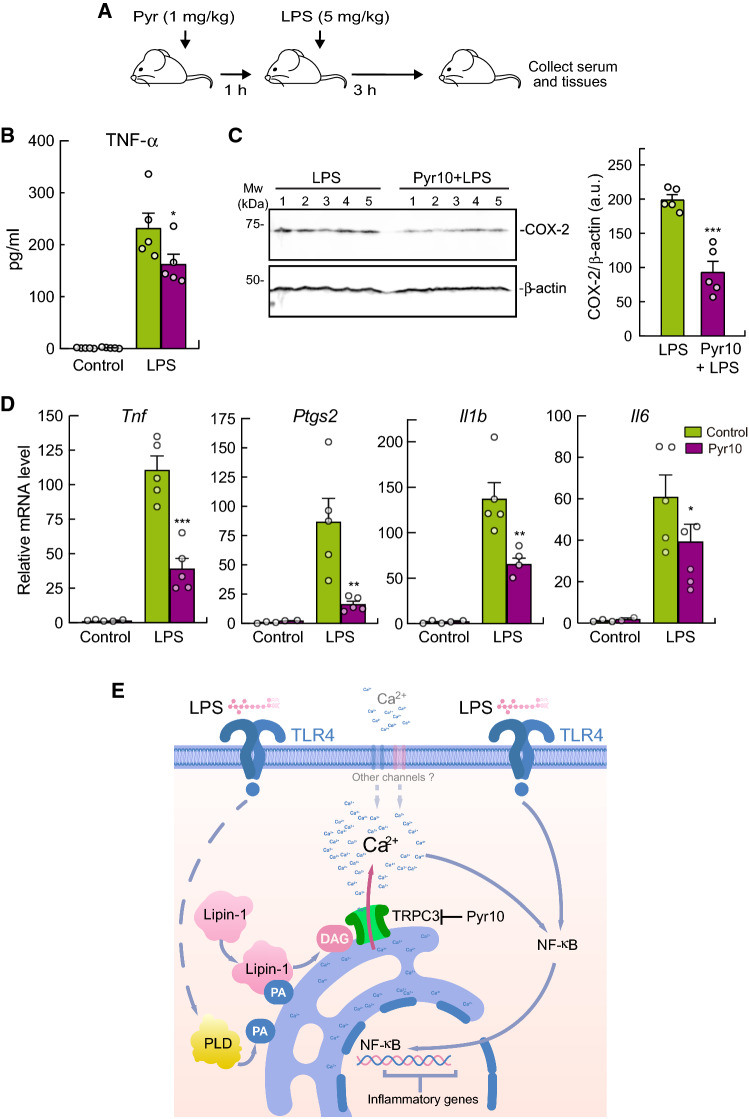
Pyr10 treatment reduces LPS-induced inflammation in mice. **A** Scheme of Pyr10 treatment and LPS-induced inflammation. Mice were intraperitoneally (i.p.) injected with 1 mg/kg Pyr10. One hour later they were treated (i.p.) with 5 mg/kg of LPS. Serum and livers were collected after three hours of treatment. **B** TNF-α levels present in the serum of the animals were analyzed by specific ELISA. Error bars represent SEM (*n* = 5). *, *p* < 0.05, by Student’s *t* test. **C** COX-2 levels were analyzed by immunoblot in liver homogenates using specific antibodies. β-Actin was used as loading control (left panel). Quantification of the bands is shown. Error bars represent SEM (*n* = 5) (right panel). ***, *p* < 0.001, by Student’s *t* test. **D** mRNA levels of the indicated genes were analyzed in liver by qPCR using *Gapdh* as the reference gene. Error bars represent SEM (*n* = 5). *, *p* < 0.05; **, *p* < 0.01; ***, *p* < 0.001, Student’s *t* test. **E** Proposed scheme depicting TRPC3 activation in the ER by lipin-1-derived DAG

We evaluated the pathophysiological relevance of TRPC3 *in vivo* by analyzing the effects of the inhibitor Pyr10 during an acute systemic inflammatory response induced by treating mice with LPS (Fig. 7). The animals were pretreated i.p. with Pyr10 (1mg/kg) for 1 h. Afterward, LPS was injected i.p. (5 mg/kg) for 3 h (Fig. 7A). Measurement of cytokine levels in blood serum showed decreased levels of TNF-α (Fig. 7B). COX-2 protein levels were assayed by immunoblot in liver, where LPS has a very potent effect. The results showed that the enzyme was less expressed in the Pyr10-pretreated group, implying reduced production of inflammatory eicosanoids under these conditions (Fig. 7C). Also, analysis of mRNA levels for different inflammatory genes showed that the drug strongly prevented the upregulation of *Ptgs*, *Tnfa*, *Il1b*, and *Il6* mRNA levels in the liver (Fig. 7D). These results demonstrate that inhibition of TRPC3 with Pyr10 reduces the inflammatory response induced by LPS in animals.

## Discussion

The involvement of Ca^2+^ fluxes in LPS signaling in macrophages and their relevance to mount a full inflammatory reaction have long been recognized [26]. However, the molecular players regulating these processes remain poorly defined. In the present work, we describe a new key effector for LPS-triggered Ca^2+^ signaling, the ion channel TRPC3. Our data support a scenario during macrophage activation by LPS wherein lipin-1 hydrolyzes the PA pools present in ER membranes, generating DAG (Fig. 7E). The ER-associated TRPC3 pools that are close to lipin-1-derived DAG thus become activated, thereby opening a pore that allows Ca^2+^ release from the organelle to the cytosol. In this manner, TRPC3 participates in the release of Ca^2+^ from intracellular stores and triggers a cascade of events that impact on NF-κB translocation to the nucleus and upregulation of inflammatory genes. Since the development of new pharmacological strategies that use TRPC3 as a target are well advanced, and we have observed that selective inhibition of TRPC3 reduces inflammation in a murine sepsis model, our study provides the foundation for new therapies against inflammatory-based diseases using drugs targeting this channel.

A striking feature of TRPC3 channels is their dependence on DAG. Regulated increases in DAG levels provide an efficient means to turn on the activity of TRPC3 in accordance with the activation state of the cells [15, 27], facilitating its role in modulating cellular Ca^2+^ fluxes. It is generally accepted that phosphoinositide-specific phospholipase C, by producing inositol 1,4,5-trisphosphate (InsP_3_) and DAG, impacts as a double-edge sword on intracellular Ca^2+^ signaling [19]. InsP_3_ interacts with specific receptors in the ER favoring Ca^2+^ release, and DAG activates TRPCs at the plasma membrane, allowing Ca^2+^ entry from the extracellular medium [19]. However, the unanticipated findings that Pyr10-sensitive ion currents are not altered by LPS at the plasma membrane level, and that TRPC3 is mainly present in intracellular membranes in macrophages, makes it difficult to envision a mechanism for channel activation coupled to phosphoinositide hydrolysis at the plasma membrane. Rather, our results are consistent with the notion that intracellular TRPC3 is activated by DAG generated close to the channel, which allows the release of Ca^2+^ from internal stores.

Considering possible candidate enzymes that could be involved in the regulation of intracellular DAG levels during TLR4 challenge, lipin-1 fulfills key requirements. For example, although lipin-1 does not reside permanently in the ER, it is able to translocate to ER membranes via a polybasic domain that facilitates binding to its PA substrate [14]. Importantly, lipin-1 has previously been found to regulate cellular DAG levels during macrophage activation by LPS and, in this manner, impact on many cellular and biological processes of activated macrophages [5, 33]. However, the molecular mechanisms governing these effects were not identified. The discovery that lipin-1 regulates TRPC3 activation and associated Ca^2+^ suggests that this phosphatase plays a wider role than anticipated during macrophage activation. From a signaling point of view, lipin-1 resembles the effects triggered by phospholipase C at the plasma membrane, i.e., promoting the accumulation of DAG and initiating Ca^2+^ fluxes. Thus, our data support the view that LPS triggers similar processes at two different cellular locations, i.e., the plasma membrane and at the ER, generating DAG to activate signaling effectors and, by different mechanisms, regulating the release of Ca^2+^ from the organelle.

Consistent with the above scenario, ER lipidomics performed in TLR4-activated macrophages has revealed that the organelle undergoes a profound lipid remodeling [1]. Interestingly, a few molecular species of PA increase in this compartment, namely PA(32:1), PA(34:1), PA(36:0), PA(40:3), and PA (40:6). It is clear that some of these species may serve as substrates for phospholipid synthesis—a process that is known to occur during TLR4 activation—but others could participate in signaling either directly or via conversion to other signaling lipids such as DAG [13]. We have shown here that PLD inhibition decreases Ca^2+^ fluxes during LPS stimulation. Macrophages express two phopsholipase D forms, called PLD1 and PLD2, and both have been found to be activated by LPS ([21, 24, 42]). While PLD1 is present at the plasma membrane and intracellular vesicles, PLD2 seems to localize at perinuclear regions [21]. Importantly, it has recently been described that during activation of macrophages by LPS, PLD1, but not PLD2, associates with TLR4 and MyD88, increasing its activity [20]. It is thus plausible that the TLR4/MyD88/PLD1 axis mediates increases in PA pools that serve as substrates for lipin-1 during the events of macrophage proinflamatory activation and sepsis development described in this study.

The participation of TRPC3 in the release of Ca^2+^ during LPS challenge does not preclude the involvement of InsP_3_ receptors as well, as proposed by previous work [10]. It should be noted that the InsP_3_ antagonist utilized in those studies, 2-aminoethoxydiphenyl borate, has also been found to block TRPC3 [8], which makes it difficult to estimate the actual participation of  InsP_3_ receptors to the LPS response. Based on our studies using ER-tagged cameleon genetic probes where TRPC3 was silenced or inhibited, we estimate that TRPC3 contributes by no less than 75% of the LPS-induced ER Ca^2+^ release. Of note, TRPC3 possesses a highly conserved InsP_3_ receptor binding region in its cytoplasmic C-terminus and, in fact, TRPC3 has long been described to work in association with IP3 receptors in other cell systems [48]. Future work should be directed toward determining whether TRPC3 associates with InsP_3_ receptors in ER membranes and collaborates to Ca^2+^ release during LPS activation of the macrophages.

To conclude, we have uncovered the ion channel TRPC3 as a new early player in LPS-stimulated responses in human macrophages. Our work introduces the concept that intracellular, lipid-activated TRPC3 participates in Ca^2+^ signaling during LPS-driven inflammation and sepsis development. Furthermore, we have presented evidence that lipin-1 provides the DAG used for TRPC3 activation in this context. These findings help fill some gaps in our perception on how innate responses are mounted. Also, due to the recent development of photopharmacology and optochemical genetics of TRPC3 that could be the foundation for novel therapeutics [25], these discoveries highlight pharmacological targeting of TRPC3 as an attractive new strategy for the treatment of sepsis and inflammatory diseases.

## Materials and methods

### Cells

Human blood monocyte-derived macrophages were prepared from buffy coats of healthy volunteer donors (Centro de Hemoterapia y Hemodonación de Castilla y León, Valladolid, Spain) as previously described [9, 29]. Non-adherent mononuclear cells were differentiated to macrophages by culture in RPMI 1640 supplemented with 2 mM L-glutamine, 100 U/ml penicillin, 100 μg/ml streptomycin, and heat-inactivated 5% human serum without exogenous cytokines for 2 weeks [9, 29].

The human promonocytic cell line THP-1 was maintained in RPMI 1640 medium supplemented with 10 mM HEPES, 10% FBS, 100 U/ml penicillin, 100 μg/ml streptomycin, 2 mM glutamine, 1% sodium pyruvate, 1% non-essential amino acids solution and 50 μM β-mercaptoethanol at 37 °C in a 5% CO2 humidified incubator. For differentiation to a macrophage phenotype, the cells were treated with 25 ng/ml PMA for 24 h, after which they were left to rest for 48 h.

Mouse peritoneal macrophages were obtained as previously described [33]. Briefly, the peritoneal cavity was flushed with 5 ml ice-cold PBS. Cells were then centrifuged at 300 g for 10 min and allowed to adhere to glass coverslips overnight. Non-adherent cells were washed away with PBS.

HEK293 cells expressing human TLR4/MD2/CD14 (HEK-TLR4, Invivogen, Catalog #293-htlr4md2cd14) were cultured in DMEM supplemented with 2 mM glutamine, 10% fetal bovine serum (FBS), 1% sodium pyruvate, 1% non-essential aminoacids solution, 100 U/ml penicillin, 100 μg/ml streptomycin, 5 μg/ml blasticidin and 25 μg/ml hygromycin B at 37°C in a 5% CO2 humidified incubator. The cells were passaged twice a week by detachment with 1 mM EDTA in PBS.

### Animals

BALB/cJ mice were obtained from the University of Valladolid Animal House (10–12-week-old males). The mice were intraperitoneally injected with the TRPC3 specific inhibitor Pyr10 (1 mg/kg) (Sigma-Aldrich) for 1h and then treated with LPS at a sublethal dose of 5 mg/kg (i.p.). For the analysis of proinflammatory factors, animals were sacrificed by ketamine (100 mg/kg) / xylacine (10 mg/kg) administration and cervical dislocation 3 h after LPS treatment. Blood was collected through cardiac puncture. Livers were collected in RNAlater (Ambion) for further analysis by real-time PCR or in protein lysis buffer for western blot analysis. Serum from animals was used for quantification of TNF-α by specific ELISAs (Thermo) following the manufacturer’s instructions. BALB/cByJ- Lpin1fld/J mice carrying a spontaneous mutation in the *Lpin1*gene (fatty liver dystrophy, *fld*) were also used [33]. All procedures involving animals were carried out under the supervision of the Institutional Committee of Animal Care and Usage of the University of Valladolid (Approval No. 7406000), and are in accordance with the guidelines established by the Spanish Ministry of Agriculture, Food, and Environment, and the European Union.

### Plasmids and mutagenesis

Human TRPC3 (eYFP-hTRPC3) and the N-terminal fragment of hTRPC3 (hTRPC3(1- 321), eYFP-N-ter-TRPC3) expression plasmids were kindly provided by Dr. K. Groschner (University of Graz, Austria). Wild-type hTRPC3 was mutagenized within the transmembrane pore domain (S6 helix) to modify its DAG discrimination capacity [27] by replacing Gly652 with Ala (G652A) using the Quick-Change XL site-directed mutagenesis kit (Stratagene, La Jolla, CA, USA) and the oligonucleotides described in Supplemental Table 1. Mutagenesis was confirmed by sequencing. The plasmids pcDNA3-Daglas-pm1 and pcDNA3-Daglas-em1 were provided by Dr. M.J. Caloca from our institute (originally from Dr. Y. Umezawa, University of Tokyo, Japan). Daglas-em1 was amplified by PCR adding 5´Xba-I and Sal-I 3’specific restriction sites and which removes copGFP gene in the plasmid inserted into lentiviral vector pCDH-CMV-MCS-EF1α-copGFP. pBudCE4.1-vYC4er (vYC4er cameleon) was provided by Dr. M.T. Alonso from our institute (originally from Dr. W. Graier, University of Graz, Austria). Construct expressing lipin-1-mCherry was amplified by PCR adding 5´Xba-I and Sal-I 3´specific restriction sites and inserted into lentiviral vector pCDH-CMV-MCS-EF1α-copGFP (System Biosciences) after removing an internal Sal-I restriction site by mutagenesis.

### Gene silencing and plasmid transfection

Gene silencing and plasmid transfection (vYC4er cameleon) of human monocyte-derived macrophages and THP-1 macrophages was achieved using a Nucleofector device (Amaxa), following the specifications of the manufacturer. Briefly, the human macrophages were harvested by treatment with 1 mM EDTA in PBS for 30 min followed by trypsin treatment for 20 min and then by gentle scraping. The THP-1 macrophages were detached by accutase treatment (Gibco) for 30 min. Cells were then washed and resuspended in 100 μl Nucleofector solution plus 1 μg plasmid or 20 nM siRNAs (Sigma). Nucleofection was carried out using the program Y-010, and the cells were resuspended in RPMI 1640 medium supplemented with 100 U/ml penicillin, 100 μg/ml streptomycin, 2 mM glutamine, and 5% heat-inactivated human serum. Cells were used for experimentation 48 h later.

Gene silencing of HEK-TLR4 cells, was carried out using Lipofectamine RNAiMAX (Thermo Fisher Scientific) as specified by the manufacturer, and experiments were conducted 48 h later. Plasmid delivery (eYFP-TRPC3, eYFP-N-ter-TRPC3, eYFP-G652A- TRPC3, and vYC4er cameleon) in HEK293-TLR4 cells was done using linear polyethylenimine 25K (Polysciences) complexed with DNA in a 2:1 ratio, respectively, for 24–48 h. Stable transfections of eYFP-TRPC3 were carried out by culturing the cells in the presence of 0.5 mg/ml G418.

### Lentiviral transduction

VSV-G–pseudotyped lentiviral particles were produced by cotransfection of 293FT cells with transfer constructs (Daglas-em1 and lipin-1-Cherry) and the compatible packaging plasmids pMD2.G and psPAX2 in the presence of PEI 25K. Viruses were harvested at 48 and 72 h after transfection. Lentiviral transduction of primary human macrophages THP-1 macrophages and HEK-TLR4 cells, were carried out using concentrated lentiviral particles in the presence of 8 μg/ml Polybrene (Sigma-Aldrich), and infected cells were selected by FACS sorting and subjected to imaging experiments.

### ***Cytosolic Ca***^***2***+^***measurement***

Cells were loaded with 3 μM Fluo-4-AM for 20 min in culture medium at 37 °C in a 5% CO_2_ incubator. Cells were then washed in indicator-free medium to remove any dye that was nonspecifically associated with the cell surface, and then incubated for a further 20-min period to allow for complete hydrolysis of the acetoxymethyl esters. Live cell fluorescence was monitored by confocal microscopy (TCS SP5X, Leica) with a HCX Plan Apo CS 40X/1.25 NA oil or with a HCS Plan Apo CS 63X/1.4 NA oil immersion lenses, using 488 nm laser excitation and an emission window of 500–560 nm, with the pinhole fully open. Before imaging started, medium was replaced by HBSS supplemented with 10 mM HEPES, with 1.3 mM CaCl_2_ and 1.3 mM MgCl_2_. We recorded a 5 min baseline before adding LPS acquiring images each 15 s. In some experiments, calibration of Ca^2+^ levels was achieved at the end of each experiment as previously described by Kao et al. [22]. Cells were treated with MnCl_2_ at a final concentration of 2 mM in the presence of 2.5 µM A23187, and then cells were lysed with 0.05% digitonin to obtain the fluorescence background signal. Fluorescence data were analyzed using a combination routine of ImageJ and Cell Profiler semiautomated custom pipeline.

### Live cell FRET imaging

Cells expressing FRET-based sensors (Daglas or vYC4er) were plated together with a small proportion of single-color control expressing cells on glass-bottom culture dishes (MatTek) coated with poly-L-lysine (Sigma) when improved adhesion was required. The culture medium was replaced with HBSS supplemented with 10 mM HEPES, pH 7.4, 1.3 mM CaCl_2_ and 1.3 mM MgCl_2_ for confocal imaging. Cells were imaged at 37 ºC on a Leica confocal system TCS SP5X, with a HCS Plan Apo CS 63X/1.4 NA oil immersion lens, correct membrane/endomembranes localization of the Daglas sensor was confirmed by YFP imaging. For ratio imaging, CFP fluorescence was obtained by 458 nm excitation and 465–510 emission, YFP by 515 nm excitation and 525–580 nm emission and uncorrected FRET by 458 nm excitation and 525–580 nm emission. *I*_*CFP*_ and *I*_*FRET*_ were simultaneously acquired while *I*_*YFP*_ was acquired alternately. ImageJ software with customized plugins was used to correct the background from raw images and to create ratio images and export raw data to Microsoft Excel for further ratio and correction calculations. To correct for any laser power illumination inconsistency, the FRET/CFP ratio values were divided by single-color YFP expressing cells values [37], then divided by the baseline normalized FRET/CFP ratio according to the following equation:

$$nFRET\; Ratio = \left[ {\frac{{\left( {\frac{{\left( {\frac{{FRET_{t = x} }}{{CFP_{t = x} }}} \right)}}{{YFP_{t = x}^{control} }}} \right)}}{{\left( {\frac{{\left( {\frac{{FRET_{t = 0} }}{{CFP_{t = 0} }}} \right)}}{{YFP_{t = 0}^{control} }}} \right)}}} \right].$$

### Patch-clamp electrophysiology

Mouse peritoneal macrophages were plated on 12-mm coverslips, previously treated with poly-L-lysine (0.01 mg/ml), for 30 min. Patch micropipettes were made from borosilicate glass (2.0 mm OD; WPI) and double pulled (Narishige) to resistances ranging from 5 to 10 MΩ when filled with the internal solution containing: 125 mM KCl, 4 mM MgCl_2_, 10 mM HEPES, 10 mM EGTA, 5 mM MgATP, pH 7.2 with KOH. Cells were bathed in an external solution containing: 141 mM NaCl, 4.7 mM KCl, 1.2 mM MgCl_2_, 1.8 mM CaCl_2_, 10 mM glucose, and 10 mM HEPES, pH 7.4 with NaOH. Immediately after starting the recording of whole-cell currents, the external solution was switched to a solution containing: 131 mM NaCl, 10 mM TEACl, 4.7 mM KCl, 1.2 mM MgCl_2_, 1.8 mM CaCl_2_, 10 mM glucose, 100 µM BaCl_2_, 100 µM niflumic acid and 10 mM HEPES, pH 7.4 with NaOH. This solution allows to record in isolation currents mediated by TRP like channels, blocking currents through voltage-dependent K^+^ channels, inward rectifier channels and chloride channels. Cells were voltage-clamped at −40 mV. Ionic currents were recorded at room temperature using the whole-cell configuration of the patch-clamp technique. Current–voltage relationships were obtained with a 1s-ramp protocol from −100 mV to +100 mV applied every 10 s. Whole-cell currents were recorded using an Axopatch 200A patch-clamp amplifier (Axon Instruments) filtered at 2 kHz (−3 dB, 4-pole Bessel filter) and sampled at 10 kHz. Recordings were digitized with a Digidata 1200, driven by CLAMPEX 10.2 software (Molecular Devices). Electrophysiological data analyses were performed using Clampfit subroutine of the pClamp software (Axon Instruments) and with Origin 7 software (OriginLab Corporation). Current amplitude was corrected for cell size variations and expressed as current density (pA/pF), by dividing for cell capacitance values.

### Semi-quantitative PCR and qPCR

Total RNA was extracted using TRIzol reagent (Ambion). cDNA was obtained using Verso cDNA kit Reverse Transcription for RT-PCR (Thermo Fisher Scientific), following the manufacturer’s instructions. For semi-quantitative PCR forward and reverse primers specific to human TRPC3, 6, 7 and β-actin were used (see Supplemental Table S1). PCR reactions were carried out on an Eppendorf Mastercycler Personal thermal cycler using Taq polymerase (BioTools) and the following parameters: denaturation at 94ºC for 30 s, annealing at 58 ºC for 30 s, and extension at 72 ºC for 40 s. A total of 40 cycles were performed followed by a final extension at 72 ºC for 5 min. PCR products were analyzed by electrophoresis with 1.5% agarose gel and visualized by GelRed staining. Bands were intensity quantified with Image Studio Software (LiCor).

Quantitative PCR analysis was performed with an ABI7500 machine (Applied Biosystems) using specific primers, 20 ng cDNA and the PowerUp SYBR Green Master Mix (Appliedbiosystems). Relative mRNA expression was obtained using the ^ΔΔ^Ct method using *ACTB* or *Gapdh* as reference genes [47].

### Colocalization analyses

Confocal z-stacks images were deconvolved using an ImageJ parallel iterative plugin (Wiener Filter Preconditioned Landweber (WPL) method) after calculating the experimental setup point spread function (PSF) using sub resolution (0.17 μm) fluorescent beads. Colocalization was performed with JACoP plugin in ImageJ. Background levels were obtained by measuring the mean intensity of each signal outside the cells and were subtracted; negative pixel values were clipped to zero. Positive values were selected by Costes automatic thresholding, removing the bias of visual interpretation [11]. Colocalization index Manders’ *M*_*1*_ and * M*_*2*_, were calculated [31]. Manders’ split coefficients are based on the Pearson’s correlation coefficient but avoid issues relating to absolute intensities of the signal, since they are normalized to total pixel intensity [31]. These coefficients vary from 0 (non-overlaping) to 1 (100% colocalization). The index *M*_*1*_ is defined as the percentage of above-background pixels from the first channel (green) that overlap above-background pixels from the second channel (red). This index is sensitive to changes in the background but not to differences in the intensity of overlapped pixels and is suitable to apply in images with a high and clear signal to background ratio.

### Immunocytochemistry analyses

For intracellular staining of endogenous TRPC3 or NFκB-p65, cells were fixed with 4% paraformaldehyde for 20 min in LabTek II chambers and then permeabilized with 0.1% Triton X-100 for 20 min at room temperature. Cells were treated with 5% goat serum for 30 min and incubated with antibodies against TRPC3 (Alomone Labs, validated in TRPC3-deficient cells), NFκB-p65 (Cell Signaling), or SERCA (Santa Cruz) for 1 h. After washing, the cells were incubated with spectrally appropriate Alexa Fluor-conjugated Fab fragments against primary species antibodies (Molecular Probes) for 1h. After intracellular staining, nuclei were counterstained with the DNA-binding dye Hoechst 33342 (Invitrogen). All images were captured with a Leica confocal system TCS SP5X inverted microscope with a HCS Plan Apo CS 63X/1.4 NA oil immersion lens. Leica Application Suite Advanced Fluorescence software was used for the capture, and ImageJ was used for deconvolution and image presentation. Translocation was analyzed using Cell Profiler software (Broad Institute) with a custom pipeline (available upon request) that automatically detects cell boundaries and calculates the ratio between cytoplasm and nuclei fluorescence median.

### Immunoblotting

Cells or tissues were lysed in a buffer 1% Triton X-100, 50 mM Tris, pH 7.4, 150 mM NaCl, 1 mM EDTA, 1 mM EGTA, 5 mM Na_4_P_2_O_7_, 50 mM sodium β-glycerol phosphate, 270 mM sucrose, 1 mM Na_3_VO_4_, 10 mM NaF, 1 mM phenylmethyl sulphonyl fluoride and a protease inhibitor cocktail (Sigma). Cell protein was separated by 10% reducing SDS–polyacrylamide gel electrophoresis and transferred to nitrocellulose membranes. The membranes were incubated with specific primary antibodies followed by incubation with HRP-conjugated secondary antibodies and detected and quantified with a LiCor Odyssey Fc infrared imaging system.

### Quantification and statistical analysis

Statistical details of experiments are indicated in the figure legends. All data analyses were performed with Prism software (GraphPad). All data are presented as means ± standard error of the mean (SEM), indicating individual biological replicates. No statistical analysis was used to predetermine sample size. For *in vivo* experiments, animals were randomized in different treatment groups. All datasets were analyzed by unpaired two-tailed Student’s *t* test. Welch’s correction was performed when the variances were significantly different. Some data required multiple *t* test, with a false discovery rate of 1% based on two stage step up by Benjamini, Krieger and Yekutieli.

## Supplementary Information

Below is the link to the electronic supplementary material.

Supplementary file1 (DOC 47 KB)

## Data Availability

Available upon request.
