# Supplementary material for: Lipin-1-derived diacylglycerol activates intracellular TRPC3 which is critical for inflammatory signaling
Source: Cell Mol Life Sci. 2021 Nov 10;78(24):8243–60. doi: 10.1007/s00018-021-03999-0 (PMC8629864; doi:10.1007/s00018-021-03999-0)
Supplement: Supplementary file 1 — Supplementary file1 (DOC 47 KB) [file 18_2021_3999_MOESM1_ESM.doc]

Supplementary information for
Lipin-1-derived Diacylglycerol Activates Intracellular TRPC3 Which is Critical for Inflammatory Signaling

Javier Casas1,2*, Clara Meana1,3*, José Ramón López-López1,2, Jesús Balsinde1,3, and María A. Balboa1,3
Correspondence to: javier.casas@uva.es or mbalboa@ibgm.uva.es This PDF file includes:
Table S1

Key Resources Table

Mutagenesis primers	Sequence	
G652A_fwd	5´-GAAAATATTGGATACGTTCTTTATGCAATATACAATGTAACTATGGTGGTC-3´	
G652A_rev	5´-GACCACCATAGTTACATTGTATATTGCATAAAGAACGTATCCAATATTTTC-3´	
siRNA	Sequence	
TRPC3_s	5´-GCUCUUGACGAUCUGGUAUGA[dT][dT]-3´	
TRPC3_as	5´-UCAUACCAGAUCGUCAAGAGC[dT][dT]-3´	
LPIN1_s	5´- GGAGUGUCUUUGAAUAGAA[dT][dT]-3´	
LPIN1_as	5´- UUCUAUUCAAAGACACUCC[dT][dA] - 3´	
Standard PCR primers	Sequence	
TRPC3_fwd	5´- GGAAAAACATTACCTCCACCTTTCA -3´	
TRPC3_rev	5´- CTCAGTTGCTTGGCTCTTGTCTTCC -3´	
TRPC6_fwd	5´-AAGACATCTTCAAGTTCATGGTC-3´	
TRPC6_rev	5´-TCAGCGTCATCCTCAATTTCC-3´	
TRPC7_fwd	5´- TGGGTTGTATTTGGCACCTC-3´	
TRPC7_rev	5´- TGGGTTGTATTTGGCACCTC-3´	
ACTB_fwd	5´- CAGAGCAAGAGAGGCATCCT-3´	
ACTB_rev	5´- ACGTACATGGCTGGGGTG-3´	
qRT-PCR primers	Sequence	
TRPC3_fwd	5´- AGAATGACTATCGGAAGCTCTCC -3´	
TRPC3_rev	5´- GGCAAGTTTGACACGACTTAATG -3´	
ACTB_fwd	5'-ATTGCCGACAGGATGCAGAA-3'	
ACTB_rev	5'-GCTGATCCACATCTGCTGGAA-3'	
PTGS2_fwd	5'-GTGCAACACTTGAGTGGCTAT-3'	
PTGS2_rev	5'-AGCAATTTGCCTGGTGAATGAT-3'	
TNFA_fwd	5'-ATGAGCACTGAAAGCATGATCC-3'	
TNFA_rev	5'-GAGGGCTGATTAGAGAGAGGTC-3'	

IL1B_fwd	5'-ATGATGGCTTATTACAGTGGCAA-3'	
IL1B_rev	5'-GTCGGAGATTCGTAGCTGGA-3'	
IL6_fwd	5'-AAATTCGGTACATCCTCGACGG-3'	
IL6_rev	5'-GGAAGGTTCAGGTTGTTTTCT -3'	
IL12B_fwd	5'-CAGCAGTTGGTCATCTCTTGG-3'	
IL12B_rev	5'-GGTCCAGGTGATACCATCTTCT-3'	
IL23A_fwd	5'-GCCTTCTCTGCTCCCTGATA-3'	
IL23A_rev	5'-GACTGAGGCTTGGAATCTGC-3'	
Ptgs2_fwd	5'-TGAGCAACTATTCCAAACCAGC-3'	
Ptgs2_rev	5'-GCACGTAGTCTTCGATCACTATC-3'	
Tnfa_fwd	5'-ACGGCATGGATCTCAAAGAC-3'	
Tnfa_rev	5'-AGATAGCAAATCGGCTGACG-3'	
Il1b_fwd	5'-GCAACTGTTCCTGAACTCAACT-3'	
Il1b_rev	5'-ATCTTTTGGGGTCCGTCAACT-3'	
Il6_fwd	5'-TAGTCCTTCCTACCCCAATTTCC-3'	
Il6_rev	5'-TTGGTCCTTAGCCACTCCTTC-3'	
Gapdh_fwd	5'-AGGTCGGTGTGAACGGATTTG-3'	
Gapdh_rev	5'-TGTAGACCATGTAGTTGAGGTCA-3'	
Antibodies	Source	Identifier/RRID	
Rabbit Monoclonal NF-κB p65 (clone D14E12) XP	Cell Signaling Technologies	#8242/AB_10859369	
Rabbit polyclonal TRPC3	Alomone Labs	#ACC-016/AB_2040236	
Mouse monoclonal SERCA2 (IID8)	Santa Cruz	#sc-53010/AB_630230	
F(ab')2-Goat anti-Rabbit IgG (H+L) 2ª Antibody, Alexa Fluor 488	Thermo Scientific	A-11070/AB_142134	
F(ab')2-Goat anti-Rabbit IgG (H+L) 2ª Antibody, Alexa Fluor 594	Thermo Scientific	A-11072/AB_142057	
PE anti-human CD284 (TLR4) (clone HTA125)	BioLegend	#312805	
Mouse Monoclonal Anti-β-Actin (Clone: AC15)	Sigma-Aldrich	#A5441/AB_476744	

Goat anti-mouse IgG IRDye 800CW	Li-Cor	#925-32210/AB_2687825	
Goat anti-rabbit IgG IRDye 680RD	Li-Cor	#925-68071/AB_2721181	
TNF alpha Mouse Uncoated ELISA Kit	
Thermo Scientific	
#88-7324-22/AB_2575076	
Reagents	Source	Identifier	
E. coli Lipopolysaccharides O111:B4	Sigma-Aldrich	L2630	
PYR10	Sigma-Aldrich	SML1243	
1-Oleoyl-2-acetyl-sn-glycerol (OAG)	Sigma-Aldrich	#O6754	
FIPI	Cayman Chemical	#13563	
Propranolol	Sigma-Aldrich	#P0884	
A23187	Sigma-Aldrich	#52665-69-7	
Thapsigargin	Sigma-Aldrich	#67526-95-8	
PMA	Sigma-Aldrich	#16561-29-8	
Hoechst 33342	Invitrogen	#H3570	
Fluo-4 AM	Invitrogen	#F14201	
